# Hemothorax as the first manifestation of idiopathic pulmonary arteriovenous malformation

**DOI:** 10.22088/cjim.9.4.410

**Published:** 2018

**Authors:** Maja Crkvenac, Marko Jakopovic, Ana Hecimovic, Gordana Pavlisa, Miroslav Samarzija, Andrea Vukic Dugac

**Affiliations:** 1 Department of Internal Medicine, General Hospital Bjelovar, Bjelovar, Croatia; 2 Department of Respiratory Diseases ‘Jordanovac’, University of Zagreb, School of Medicine, Zagreb, Croatia

**Keywords:** Idiopathic pulmonary arteriovenous malformations, Pleural effusion, Hemothorax, Catheter embolization

## Abstract

**Background:**

Pulmonary arteriovenous malformations (PAVM) are rare pulmonary vascular anomalies and hemothorax as a presenting feature of PAVM is a very rare occurrence.

**Case presentation:**

A 45-year old woman presented with chest pain and breathlessness. A chest x-ray showed left-sided pleural effusion. An emergency MSCT scan with contrast showed no signs of pulmonary embolism but instead a probable AV malformation was shown. Diagnostic thoracocentesis revealed hemorrhagic exudate with negative cytology and microbiology findings. Thoracic drainage was performed resulting with complete regression of hemothorax. Three months later, patient was treated with transcatheter embolization of PAVM with good clinical outcome.

**Conclusions:**

We have shown that management of PAVM related hemothorax initially by thoracic drainage followed by later on performed catheter embolization of the PAVM could lead to a successful outcome.

Pulmonary arteriovenous malformations (PAVMs) are abnormal direct communications between pulmonary arteries and veins (1). They are mostly associated with hereditary hemorrhagic telangiectasia (HHT) (1) whereas patients who are considered to have idiopathic PAVMs have rarely been described in the literature. Although most patients are asymptomatic, PAVM are not benign lesions and they can be associated with a wide spectrum of clinical manifestations. Most of these patients present with chest pain and dyspnea and are misdiagnosed as pulmonary embolism. Hemothorax is a rare clinical entity that can be caused by heterogeneous factors including malignancies or pulmonary, vascular and hematological abnormalities. Spontaneous hemothorax as a result of a ruptured pulmonary arteriovenous malformation (PAVM) is a rare and potentially life threatening event that requires immediate interventional therapy in most cases. We report a case of spontaneous hemothorax as a result of pulmonary ateriovenous malformation treated initially with thoracic drainage followed by later on catheter embolization.

## Case Presentation

A non-smoker 45-year old woman presented with left-sided chest pain and breathlessness over 7-day duration, with no history of chest trauma. She had no signs of hemodynamic instability and did not appear to be in significant respiratory distress with only mild partial respiratory insufficiency.

## Methods

**Figure 1:**
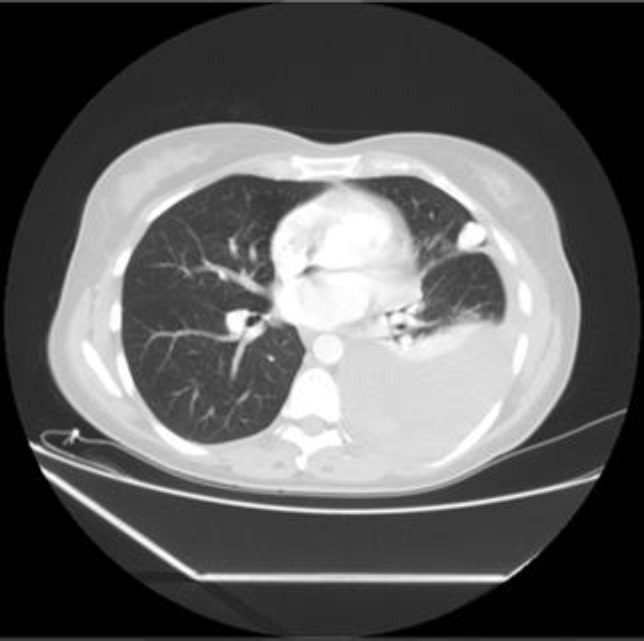
Emergency MSCT scan with contrast showed probable AV malformation 17 mm in diameter in anterior segment of left upper lobe with large pleural effusion

Laboratory findings were within normal limits apart from mild normocytic anemia (Hgb 106 g/L) and elevated D dimers (2.06 mg/L). Accordant to clinical examination of the chest, chest x-ray confirmed left-sided pleural effusion. An emergency MSCT scan with contrast showed no signs of pulmonary embolism but instead a probable AV malformation 17 mm in diameter was shown in anterior segment of left upper lobe (figure 1). Diagnostic thoracocentesis revealed hemorrhagic exudate in organizing stage (with protein 42 g/L, glucose 4, 4mol/L, lactate dehydrogenase 2698 U/L). Both pleural fluid cytology and microbiology findings were negative. After thoracic drainage was performed, along with antibiotic treatment, gradually regression of hemothorax occurred. Echocardiography and right heart catheterization findings were within normal limits, excluding pulmonary hypertension.

## Results

**Figure 2:**
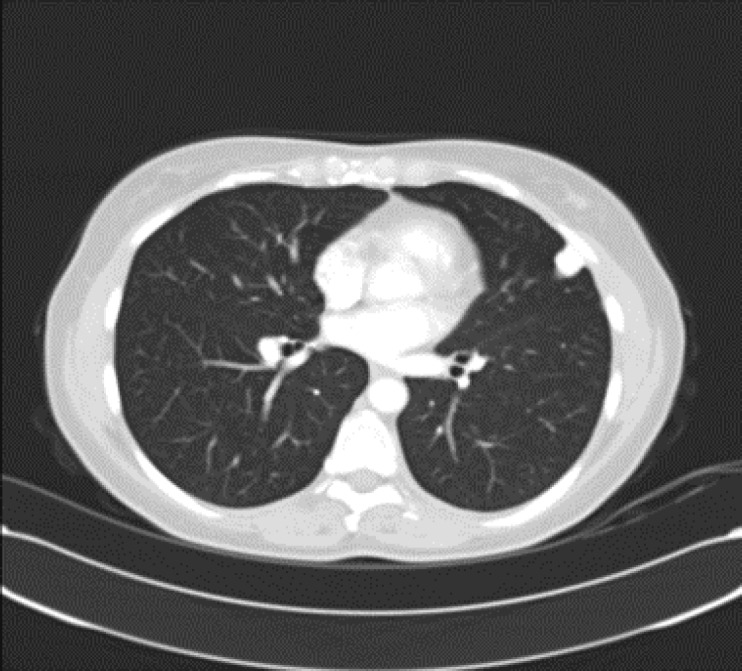
Control MSCT scan with contrast confirmed a simple AV malformation in the peripheral area of left upper lobe supplied from superior left pulmonary artery, and with a drainage vein entering the pulmonary veins and left atrium. *Complete resolution* of *pleural effusions* occurred

MSCT scan with contrast performed three months later then confirmed a simple PAVM in the peripheral lingular area supplied from superior left pulmonary artery, and with a drainage vein entering the pulmonary veins and left atrium (figure 2). The feeding artery with the diameter of 4.5-6 mm was found to be suitable for transcatheter embolization, which was afterwards successfully performed with six embolization coils (04/2014).

**Figure 3:**
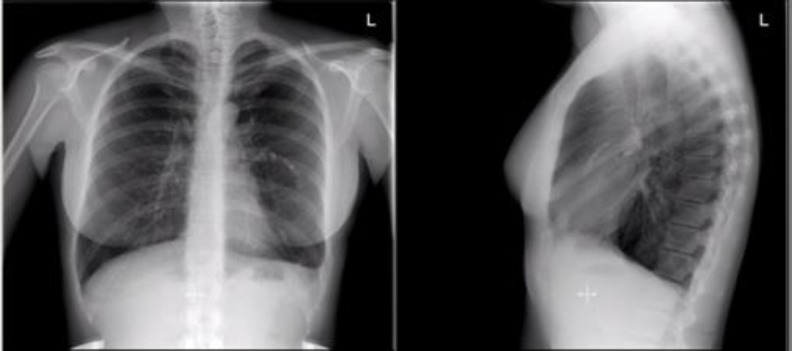
Postembolization chest x-ray showing six embolization coils with complete resolution of pleural effusion

 During further treatment, the patient participated in pulmonary rehabilitation program because of persistent dyspnea on exertion and since then has been attending regular follow-ups with normal radiological and pulmonary function test findings without recurrence of pleural effusion (figure 3).

## Discussion

Pulmonary arteriovenous malformations (PAVMs) are rare abnormal communications between the branches of pulmonary arteries and veins (2, 3). They occur more frequently in women and most PAVMs (80-95%) are associated with hereditary hemorrhagic telangiectasia (HHT) (1, 3). In a series of 219 consecutive PAVM patients, a clinical diagnosis of HHT could be established in 93.6% of cases (2). PAVMs can also occur in a variety of acquired medical conditions such as hepatic cirrhosis, chest trauma or surgery, mitral stenosis, schistosomiasis, actinomycosis, Fanconi's syndrome, metastatic thyroid carcinoma. The remainder are presumed to be idiopathic (1,4). According to reviewed literature, most PAVM studies were largely comprised of HHT patients. First study of clinical and imaging characteristic of idiopathic PAVMs (1) showed that they are anatomically similar to HHT-related PAVMs except for a greater number of solitary PAVMs and a lack of lower lobe predominance. 

The clinical manifestations and complications of idiopathic PAVMs are also similar to those associated with HHT (1). More than half of patients with PAVM are asymptomatic, and lesions are often detected by routine chest x-ray (5). In most cases, symptoms related to PAVMs typically begin during the fourth through sixth decade of life, whereas, symptoms of HHT frequently develops before the age of 20. The most common pulmonary symptoms of PAVMs are dyspnea and hemoptysis, as well as those attributable to underlying HHT. Neurological complications include transient ischemic attack, cerebral stroke or abscess due to paradoxic embolization facilitated by right-to-left shunting (6). In our case of a solitary PAVM, on the basis of absent HHT related clinical features, negative family history as well as the age of onset, HHT can be ruled out.

Potentially life-threatening complications that occur in less than 10-20 percent of patients with PAVMs are hemothorax and hemoptysis (7). Hemothorax is a clinical entity that in most cases can be caused by trauma, coagulopathy, pneumothorax, neoplasms, pleural endometriosis and vascular anomalies. Spontaneous hemothorax as a complication of a PAVM is a very rare and potentally dangerous (7). Ali et al. found only 32 reported cases of hemothorax associated with PAVM till 2008 and half of these cases had documented HHT.(8). In a series of 143 patients referred for transcatheter embolotherapy, 8% had a hemothorax requiring hospitalization and massive hemorrhage was the presenting symptom in 9 of the 11 subjects (9). Worsening of PAVM with pregnancy has been reported by multiple studies, and approximately 30% of massive hemorrhagic events occur during pregnancy (10). Pregnancy can increase the size of PAVMs, due to increased cardiac output and hormonal effects on the blood vessels resulting with rupture of a PAVM and hemothorax.

While hemoptysis is likely due to rupture of either parenchymal PAVM or an endobronchial teleangiectasia, hemothorax results from rupture of a subpleural PAVM. As seen in our patient, probable rupture of PAVM in peripheral area led to this rare complication. Massive hemoptysis and massive hemothorax from rupture of PAVM into the pleural space have been reported, with fatal outcomes (9), but hemothorax that is not life-threatening as in our case report is not described in the literature. The classic roentgenographic sign of PAVM is a round or oval sharply- defined mass of uniform density, often lobulated, mostly in lower lobes of the lung. However the shadow may be hidden by hemothorax as seen in our case (figure 1). 

 Contrast-enhanced pulmonary angiography remains the gold standard for defining the anatomy of PAVMs that are considered potentially suitable for embolotherapy, which has largely replaced surgical intervention. PAVMs were historically treated with surgical resection. As endovascular techniques developed, embolization became the mainstay of treatment. Initial management with therapeutic embolization may well be appropriate in most patients who present with symptomatic PAVM. In patients who are hemodynamically unstable or in whom embolization has failed, open surgical resection of the PAVM has been successful (5). Treatment with embolotherapy is suggested for patients with one or more PAVM with a feeding artery diameter of more or equal to 2-3 mm, in case of progressive enlargement PAVM, paradoxic embolization and symptomatic hypoxemia. The technique of coil embolotherapy involves the localization of the pulmonary arteriovenous malformation with angiography, followed by selective catheterization of the feeding artery. A steel coil is advanced through the catheter and placed distal to any branch of the vessel. Sometimes, more than one coil is required to completely occlude the vessel as it was in our case. 

 Transcatheter embolotherapy has proven to be a safe and effective treatment for sporadic PAVMs providing excellent functional and radiological improvement (3, 4), as seen in our case. Due to the rarity of idiopathic PAVMs, especially presenting with hemothorax, there are only limited reports on their treatment outcomes. Most reported cases of hemothorax associated with PAVMs so far have been related to HHT (6). 

In reviewed cases, which included mostly acute onset of PAVM associated with hemothorax, primary treatment was surgical or urgent embolization interventions, or combination of both (6, 11, 12, 13). To our knowledge, there are no reports on hemothorax associated with idiopathic PAVM being managed as it was in our case; initially by thoracic drainage and later on performed transcatheter embolization of the PAVM. In our experience, the sequence of these procedures in a respiratory and hemodynamically stable patient led to a successful outcome.

In conclusion PAVMs are relatively rare disorders with very common presentations and they are an important part of the differential diagnosis of pulmonary problems like hemoptysis, pulmonary nodule and hypoxemia. Although rarely seen, a PAVM as underlying cause of a spontaneous hemothorax should also be considered.  Contrast-enhanced computed tomography is a valuable and widely available diagnostic tool for patients with abnormal chest radiography suspicious of PAVM. There is little data on idiopathic PAVMs in the literature, especially presenting with hemothorax, and hopefully this case could benefit treatment of some of PAVM patients, which is why we consider our case worthy of reporting.

We have shown that management of PAVM-related hemothorax initially by thoracic drainage followed by later on performed catheter embolization of the PAVM in a low risk patient could lead to a successful outcome.
